# Human brain computing and brain-inspired intelligence

**DOI:** 10.1093/nsr/nwae144

**Published:** 2024-04-22

**Authors:** Jianfeng Feng, Viktor Jirsa, Wenlian Lu

**Affiliations:** Institute of Science and Technology for Brain-Inspired Intelligence, Fudan University, China; Key Laboratory of Computational Neuroscience and Brain-Inspired Intelligence (Fudan University), Ministry of Education, China; Shanghai Center for Mathematical Sciences, Fudan University, China; Department of Computer Science, University of Warwick, UK; Zhangjiang Fudan International Innovation Center, China; Institut de Neurosciences des Systèmes, Faculté de Medécine, Aix Marseille Université, France; Institute of Science and Technology for Brain-Inspired Intelligence, Fudan University, China; Key Laboratory of Computational Neuroscience and Brain-Inspired Intelligence (Fudan University), Ministry of Education, China; Shanghai Center for Mathematical Sciences, Fudan University, China

The relationship between structure, neural activity dynamics and intelligent human brain function is complex and multifaceted with deep roots in several academic disciplines. Its understanding is a fundamental goal of brain research [1] and may indicate the way towards the next generation of artificial intelligence [2]. There are major challenges on the way, several of which are addressed in this Special Topic of *National Science Review*.

First, establishing a computational neuronal network model of the human brain is extremely difficult due to its size, neuromorphic details and topological structure. The human brain is an immensely complex organ, with 86 billion neurons and 100 trillion synapses, which overshadows all man-made attempts at intelligence, such as large language models. Mathematically modeling the detailed structure and connectivity of the brain at various scales ranging across several orders is a formidable task. The question arises as to what degree the multiscale architecture is necessary to support brain function. As a consequence, mapping the entire connectome (a comprehensive map, comprising all neural connections) is a priority and deciphering the function of each brain region remains significantly challenging [3].

At the mesoscale, Maass [4] presented the essential principles for cortical microcircuits, which appear to allow the understanding of cortical computations and are likely to yield computing capabilities that are substantially more brain-like and more energy-efficient. At the microscale, Senden *et al.* [5] reviewed hybrid integrative brain models. These integrate the complex interactions between the brain, body and environment in a modular way, incorporating global processes, such as attention, that are potentially mediated via hub structures such as the thalamus. To advance the analysis of such interactions, Vohryzek *et al.* [6] proposed a Harmonic Decomposition of Spacetime framework for functional magnetic resonance imaging, based on eigenfunctions of the normalized Laplacian of the voxel-based structural matrix. In psychedelics, these authors identified significant harmonic modes and functional harmonic changes.

Secondly, neuromorphic computation takes a constructive ‘build-to-understand’ approach in addressing the structure–function dichotomy of the brain, and aims to design and implement computing systems that are inspired by the architecture and functioning of the human brain. Replicating the complexity of neural networks, at the size and real-time capability of the human brain, requires the development of hardware that can accurately emulate the parallel and distributed nature of neural processing and has an energy efficiency that is comparable to that observed in the brain [7]. Designing and fabricating neuromorphic chips is the unique pathway to achieving neural networks with the scale and complexity of the human brain.

In particular, Darwin3 [8] is the current release of a neuromorphic-specific instruction set and high-density connectivity storage technology. It leads to an upgrade in the number of neurons to >2 million, with impressive model capabilities and learning programmability. The recent focus of neuromorphic systems has been mostly towards brain science. However, such systems are now having their scope extended to Artificial General Intelligence with engineering applications. One noteworthy instance of this paradigm is the Hybrid Neural Network (HNN), which integrates computer science-oriented artificial neural networks with neuroscience-oriented spiking neural networks [9]. The main characteristic of the new generation of neuromorphic systems is to support HNN. For instance, the second generation of SpiNNaker (SpiNNaker2) has more than an order of magnitude improvement in functional density and energy efficiency over the first generation and supports HNN, leading towards energy-efficient cutting-edge artificial intelligence. Even sparse event-based large language models are looming ever closer [10].

Thirdly, clinical applications employing computational models of the human brain drive are at the forefront of brain science and require the development of novel efficient scientific methodologies. The digital twin brain concept and technology were introduced to produce a digital representation of the structure, computing processes and intelligent functions of a biological brain [11]. This will provide a platform for digitally testing diverse cognitive and medical approaches that cannot be carried out on a real biological subject. Clinical applicability, however, necessitates that the digital representation is subject-specific. The Virtual Brain Twin presents such a personalized whole-brain network model that is informed by an individual's own brain-imaging data. The approach includes spatial masks for relevant parameters across five brain disorders and demonstrates their use based on physiological and pathophysiological hypotheses [12]. The digital twin brain is based on a spiking neuronal network, on the scale of the whole brain, with ≤20 billion neurons and a data-constrained structure. Distinguished by a reverse engineering approach, it provides a paradigm for assimilating tasks. In this way, the authors have found that the closer the brain is, in both scale and architecture, the greater the similarity between the simulated model and the real brain—in both the resting state and during tasks. This exposes to a certain degree (and for the first time) the necessity for aiming to reproduce the scale and structural details of the human brain in a computational model, in order to establish biologically sound brain-inspired intelligence [13].

In summary, integrating models, data and dedicated computability reaches the fundamental principle of brain computing. For instance, the free-energy principle entails the Bayesian brain hypothesis that can be implemented by the many schemes considered in these fields [14]. The Bayesian mechanics of brain computing gives a unique route to understanding authentic (neuromimetic) intelligence and, more importantly, points towards the development of brain-inspired intelligence.

***Conflict of interest statement.*** None declared.

## References

[1] Jain V . Nature 2023; 623: 247–50. 10.1038/d41586-023-03426-3 37935967

[2] Savage N . Nature 2019; 571: S15–7. 10.1038/d41586-019-02212-4 31341311

[3] Feng JF . Quant Biol 2023; 11: 471–3. 10.1002/qub2.6 PMC12806899 41675532

[4] Maass W . Natl Sci Rev 2024; 11: nwad301. 10.1093/nsr/nwad301 38577672 PMC10989294

[5] Senden M, van Albada SJ, Pezzulo G et al. Natl Sci Rev 2024; 11: nwad318. 10.1093/nsr/nwad318 38577673 PMC10989280

[6] Vohryzek J, Cabral J, Timmermann C et al. Natl Sci Rev 2024; 11: nwae124. 10.1093/nsr/nwae124 38778818 PMC11110867

[7] Schemmel J, Brüderle D, Grübl A et al. A wafer-scale neuromorphic hardware system for large-scale neural modelling. IEEE International Symposium on Circuits and Systems, Paris: France, 30 May–2 June 2010.

[8] Ma D, Jin X, Sun S et al. Natl Sci Rev 2024; 11: nwae102. 10.1093/nsr/nwae102 38689713 PMC11060491

[9] Liu F, Zheng H, Ma S et al. Natl Sci Rev 2024; 11: nwae066. 10.1093/nsr/nwae066 38577666 PMC10989656

[10] Furber S . Natl Sci Rev 2024; 11: nwad283. 10.1093/nsr/nwad283 38577676 PMC10989295

[11] National Academies of Sciences, Engineering, and Medicine . Foundational Research Gaps and Future Directions for Digital Twins. Washington, DC: The National Academies Press. 2024. 39088664

[12] Wang HE, Triebkorn P, Breyton M et al. Natl Sci Rev 2024; 11: nwae079. 10.1093/nsr/nwae079 38698901 PMC11065363

[13] Lu W, Zeng L, Wang J et al. Natl Sci Rev 2024; 11: nwae080. 10.1093/nsr/nwae080 38803564 PMC11129584

[14] Lu W . Natl Sci Rev 2024; 11: nwae025. 10.1093/nsr/nwae025 38689714 PMC11060478

